# Local Corticosteroid Injection Versus Dry Needling in the Treatment of Lateral Epicondylitis

**DOI:** 10.7759/cureus.31286

**Published:** 2022-11-09

**Authors:** Vishnudharan Nagarajan, Prabhu Ethiraj, Arun Prasad P, Arun H Shanthappa

**Affiliations:** 1 Department of Orthopaedics, Sri Devaraj Urs Medical College, Sri Devaraj Urs Academy of Higher Education and Research, Kolar, IND

**Keywords:** elbow pain, corticosteroid injection, dry needling, lateral epicondylitis, tennis elbow

## Abstract

Background

Lateral epicondylitis (LE) is an inflammation or micro-tearing of the tendons that join the forearm muscles on the lateral aspect of the elbow. Primary treatment of LE includes rest from offending activity and corticosteroid therapy for pain control. Dry needling (DN) is a relatively new therapy for LE. This study examined the results of DN therapy with corticosteroid injection. We aimed to compare pain relief and improvements in functional disability of LE patients treated via DN and corticosteroid injection in a tertiary care center.

Methodology

A prospective randomized control study was conducted among 54 patients in the Orthopaedics Department of R L Jalappa Hospital from January 2022 to May 2022. Patients received either DN or injectable corticosteroid therapy, and treatment groups were randomized using single-blinded randomization with sealed envelopes. Patients were evaluated using the Patient-Related Tennis Elbow Evaluation (PRTEE) score before the intervention and four and eight weeks after the intervention.

Results

A total of 54 patients were included in the final analysis. The mean age in the DN group was 43.96 ± 8.15 years and 44.74 ± 8.33 years in the corticosteroid group. In the DN group, 17 (62.96%) patients were male, and in the corticosteroid group, 16 (59.26%) patients were male. The differences in the PRTEE score at the fourth and eighth-week follow-up with baseline value (pre-injection) were statistically significant (p < 0.001).

Conclusions

DN is a low-cost, minimally invasive, and low-risk therapy whereas corticosteroid therapy is costly and produces systemic side effects in the long term. In this study, during the last follow-up visit, the PRTEE score improved in the DN group compared to the corticosteroid group.

## Introduction

Lateral epicondylitis (LE) is an excruciating condition of the elbow wherein there is inflammation or micro-tearing of the extensor tendon origin of the forearm muscles at the outer side of the elbow. The extensor origins of the forearm muscles become damaged because of overdoing and repeating similar actions. As a result, it causes the elbow joint’s lateral region to become painful, especially on exertion [1].

LE was initially described in 1873 [2]. The pain of LE is felt typically where the tendons of the forearm muscles, particularly the extensor carpi radialis brevis (ECRB), originate at the lateral epicondyle.

The overall prevalence of LE ranges from 1% to 3% of the total population affecting both genders equally [3]. It is a musculoskeletal condition that scrapes adulthood with pain and disability. In the general population, hazards for the development of this condition include smoking, being overweight, and performing repetitive motions with vigorous activity involving the elbow, forearm, and wrist for at least two hours each day.

The principal mechanism is due to a degenerative overdoing movement of the ECRB along with the common extensor tendon. Clinical signs include a point of soreness distal to the lateral epicondyle at the site of the ECRB insertion, decreased grip strength, and positive results on provocative tests such as the Cozen’s and Mills. It is important to rule out any further differential diagnoses that overlap clinically, including cervical radiculopathy, posterolateral rotatory instability, radial tunnel syndrome, etc. The histological findings include the proliferation of tissue granulation, micro-rupture, an abundance of fibroblasts, vascular hyperplasia, amorphous collagen, and a prominent absence of traditional inflammatory cells within the skin. Previously, the term was described as angiofibroblastic dysplasia based on multiple histologic studies unfolding its microscopic appearance and characteristics [4,5].

The primary management of LE includes rest from the offending activity. Pain control options also include applying ice after exercise, using oral or topical non-steroidal anti-inflammatory drugs, and using corticosteroid therapy. Forearm counterforce straps are recommended to control stiffness. To ease the strain on the wrist extensors, a cock-up wrist splint may be given [6]. Basic treatment might not work to resolve the complaints of several patients, and secondary therapies, which are generally invasive, are prescribed in such scenarios [7,8]. Dry needling (DN) is still a relatively novel treatment for LE, despite having been used to treat trigger points, myofascial pain, and rotator cuff issues.

Prior work on DN therapy demonstrated it to be a safe and efficient approach to treating LE [9]. In this research, the efficacy of DN is compared with injectable corticosteroids, which is established in previous studies as an effective treatment modality for LE management.

## Materials and methods

This prospective randomized control trial was carried out among 54 patients in the Orthopaedics Department of R L Jalappa Hospital from March 2022 to June 2022 after receiving approval from the institutional ethics committee of Sri Devaraj Urs Medical College, Tamaka, Kolar (approval number: DMC/KLR/IEC/ 689/2021-22). The study comprised patients between the ages of 20 and 75 who were clinically diagnosed with LE utilizing provocative tests and point tenderness at the insertion of the ECRB at the lateral epicondyle. A minimum of three weeks of abstinence from the offending activity along with analgesic medication were given to the study participants before the start of the study. Patients with poor skin conditions/skin disorders and any contraindication to the procedure such as bleeding disorders, poor glycemic control, and inflammatory arthritis were excluded. After providing their signed consent for the research, participants underwent either DN or injectable corticosteroid therapy. Treatment groups were randomized via single-blinded randomization with closed envelopes.

In the DN group, 8-12 disposable filiform needles of size 25 mm were inserted under aseptic conditions at the lateral epicondyle region, close to the site of maximal tenderness, for approximately 10-12 minutes down to the bone without any local anesthesia. Participants received five sessions in total, twice a week from a single therapist. In the corticosteroid group, participants received a single dose (2 mL) of triamcinolone acetate (40 mg/mL) injection. During the trial, the participants did not undergo any other treatments or take analgesics for LE. The participants were assessed using the Patient-Related Tennis Elbow Evaluation (PRTEE) before and four and eight weeks after the intervention. After the last session of DN, the first post-therapy evaluation in the DN group was conducted.

Data collection and analysis

Data were entered into MS Excel and analyzed using SPSS software version 22 (IBM Corp., Armonk, NY, USA) [10]. PRTEE scores (pre-injection, fourth-week follow-up, and eighth-week follow-up) were considered primary outcome variables. Drug (DN vs. corticosteroid) was considered the primary explanatory variable. Age, gender, duration, etc. were considered as other study-relevant variables. Descriptive analysis was done using the mean and standard deviation for quantitative data and frequency and percentages for qualitative variables. The normality of the quantitative data was checked using the graphical presentation in the form of histograms and normality Q-Q plots. The Shapiro-Wilk test was also conducted to assess normal distribution.

For normally distributed quantitative parameters, the mean values of both groups were compared using an unpaired t-test (two groups). The change in the quantitative parameters before and after the intervention was assessed by a paired t-test (in the case of different periods). Qualitative variables were compared and assessed between two study groups using the chi-square test/Fisher’s exact test (if the expected number in any one of the cells was <5, Fisher’s exact test was used.). A p-value of <0.05 was considered statistically significant.

## Results

In the final analysis, a total of 54 patients were included. The patients were equally distributed into the two study groups after randomization among the study population. Each group included a total of 27 patients, and pre-treatment clinical evaluation using the PRTEE score was done before the intervention. There were no shortcomings or follow-up losses in any of the patients who underwent either of the therapy.

**Figure 1:**
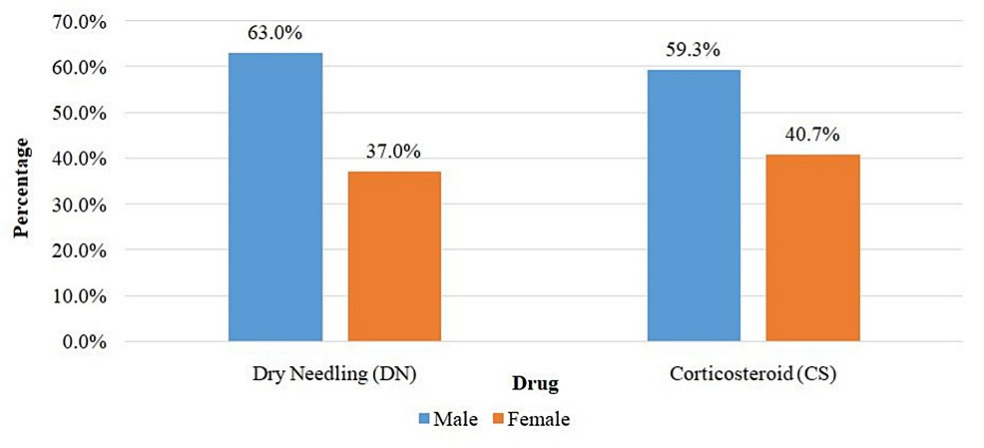
Cluster bar chart of comparison of gender between groups (N = 54).

The age distribution and sex distribution between the groups were almost comparable. The mean for age distribution in the DN group was 43.96 ± 8.15 years and 44.74 ± 8.33 years in the corticosteroid group which was insignificant statistically (p = 0.730) (Table 1). In the DN group, 17 (62.96%) were male, and the remaining 10 (37.04%) were female. In the corticosteroid group, 16 (59.26%) were male, and the remaining 11 (40.74%) were female. Therefore, no evidence of a statistically significant relationship between gender and two distinct groups was identified (p = 0.780) (Table 2, Figure 1).

**Table 1:** Comparison of mean age between groups (N = 54).

Parameter	Drug (mean ± SD)		P-value
	Dry needling (N = 27)	Corticosteroid (N = 27)	
Age	43.96 ± 8.15	44.74 ± 8.33	0.730

**Table 2:** Comparison of gender between groups (N = 54).

Gender	Drug		Chi-square	P-value
	Dry needling (N = 27)	Corticosteroid (N = 27)		
Male	17 (62.96%)	16 (59.26%)	0.078	0.780
Female	10 (37.04%)	11 (40.74%)		

**Figure 2:**
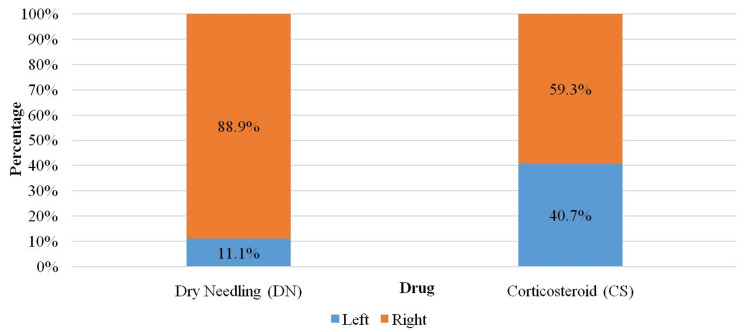
Staked bar chart of comparison of side between groups (N = 54).

In the DN group, three (11.11%) were left-sided, and the remaining 24 (88.89%) were right-sided. In the corticosteroid group, 11 (40.74%) were left-sided, and the remaining 16 (59.26%) were right-sided. Both groups of the study population exhibited right-hand dominance. No association could be established between the side and two different groups (p = 0.013) (Table 3, Figure 2).

**Table 3:** Assessment of side between groups (N = 54).

Side	Drug		Chi-square	P-value
	Dry needling (N = 27)	Corticosteroid (N = 27)		
Left	3 (11.11%)	11 (40.74%)	6.171	0.013
Right	24 (88.89%)	16 (59.26%)		

In the DN group, the mean PRTEE score before the start of therapy was 70.11 ± 4.65, and in the corticosteroid group, the pre-injection score was 64.93 ± 4.61, which was not statistically significant. Following therapy, follow-up was done at the fourth and eighth weeks using the PRTEE score, and in the DN group, it was noted to be 46.96 ± 4.43 and 38.04 ± 5.67, respectively. In the corticosteroid group, PRTEE scores at the fourth and eighth-week follow-up period were 49.19 ± 4.25 and 44.11 ± 3.45, respectively. Considering pre-therapy PRTEE scores as a baseline, the follow-up scores were compared and had a p-value <0.001, which is statistically significant (Table 4). During the therapy and follow-up periods, there were no complications in either group.

**Table 4:** Association of PRTEE scores within the groups PRTEE: Patient-Related Tennis Elbow Evaluation

Follow-up periods	PRTEE (mean ± SD)	Mean difference	95% CI of mean difference		P-value
			Lower	Upper	
Dry needling (N = 27)					
Pre-injection score	70.11 ± 4.65				
Fourth-week post-injection score	46.96 ± 4.43	23.15	20.59	25.70	<0.001
Eighth-week post-injection score	38.04 ± 5.67	32.07	29.61	34.54	<0.001
Corticosteroid (N = 27)					
Pre-injection score	64.93 ± 4.61				
Fourth-week post-injection score	49.19 ± 4.25	15.74	13.86	17.62	<0.001
Eighth-week post-injection score	44.11 ± 3.45	20.81	18.96	22.67	<0.001

## Discussion

The goal of this study was to evaluate the effectiveness of DN and corticosteroid injections. PRTEE scoring, which has been frequently utilized in research of this kind in the past, was employed to evaluate patient’s functional state both before and after the intervention [9]. In total, 54 patients who were clinically confirmed to have LE comprised the study population. Participants in the trial were randomly divided into two groups, each with 27 individuals; one group underwent treatment with DN, while the other underwent treatment with injectable corticosteroids.

The mean age of participants in the DN group was 43.96 ± 8.15 years and 44.74 ± 8.33 years in the corticosteroid group, which resembled the population sample of the studies reported by Uygur et al. [11] and Güngör et al. [12]. In this study, in the DN group, 17 (62.96%) patients were male, and the remaining 10 (37.04%) were female. In the corticosteroid group, 16 (59.26%) patients were male, and the remaining 11 (40.74%) were female. However, gender distribution was equal in the studies by Tahir et al. [13] and Brennan et al. [14]. In this study, the right-sided lateral epicondyle was most commonly affected similar to the patients in the study by Yalvac et al. in which 15.9% were affected on the left side and the remaining 84.1% were affected on the dominating right side [15]. Uygur et al. measured the magnitude of pain and disability using the Foot Function Index (FFI) [16]. The mean score of FFI at three weeks in the DN group was 27.7 ± 9.82 versus 33.6 ± 10.6 in the injectable corticosteroid therapy group. In the follow-up after six months, an improvement was seen in both groups which compared favorably to the study of Uygur et al. [11].

Although the functional evaluation scores using PRTEE scoring before the treatments were similar between the two groups, the follow-up PRTEE scores showed that the patients treated with DN exhibited significantly more functional improvement than the corticosteroid group at the fourth and eighth-week follow-ups. The outcomes in the corticosteroid group on extended follow-ups indicated that its effects are diminishing. This is consistent with the findings of earlier research that assessed the efficacy of corticosteroid treatment [17].

DN is cost-effective and has a smaller learning curve for the therapist as it is easy to perform and is less invasive. Although the complication rate following DN therapy is comparatively less compared to corticosteroid therapy, some minor local complications such as injection site pain, redness, and other transient local inflammatory reactions can be expected. Another drawback is the need for multiple sessions, such as five sessions in this study, and the loss of follow-up.

Corticosteroid Injection has the advantage that it gives functional improvement following the first injection whereas DN requires multiple sessions. Although we did not encounter any major complications following a single corticosteroid injection during the maximum follow-up of eight weeks, there are more chances of patients developing complications following multiple injections ranging from skin pigment changes to tendon atrophy and delayed wound healing, as suggested by similar previous studies with long-term follow-up and larger sample size [18].

While LE is often a self-limiting illness in several circumstances, it can become resistant in others if the patient continues to engage in the offending physical activity. As discussed all known modalities of treatment considered for LE have their pitfalls and no single modality is superior. DN has gained importance because it is safe and economical. Overall, financial concerns should always be borne in mind while considering treatment options with comparable efficacy.

Limitations

Further studies are needed to ascertain how both therapies would work in the long term because the trial was only conducted for a short time with a limited follow-up period. The accuracy of the research is constrained by the limited sample size. Even though DN was performed by a single therapist, its technique was not standardized. The validity of the study would have been expanded had other evaluation techniques been used in addition to the PRTEE scores.

## Conclusions

This study aimed to prove that DN therapy has efficacy comparable to corticosteroids in LE treatment. Considering that DN offers progressively better functional outcomes than corticosteroid therapy, it can be suggested that DN should be thought of as a feasible replacement. Though multiple sessions of DN are required, it has added advantages such as being cost-effective, minimally invasive, and having fewer complications compared to corticosteroids, despite corticosteroids being less cumbersome with the need for a single dose. To more fully evaluate the utility of DN as an effective method of managing LE, additional research with large study populations and extended follow-up is necessary.
